# The Interplay between Muscular Grip Strength and Bone Mineral Density with Consideration of Metabolic and Endocrine Parameters in Individuals with Turner Syndrome

**DOI:** 10.3390/biomedicines11123125

**Published:** 2023-11-24

**Authors:** Mariola Krzyścin, Karolina Gruca-Stryjak, Ewelina Soszka-Przepiera, Igor Syrenicz, Adam Przepiera, Aneta Cymbaluk-Płoska, Žana Bumbulienė, Elżbieta Sowińska-Przepiera

**Affiliations:** 1 Department of Reconstructive Surgery and Gynecological Oncology, Pomeranian Medical University in Szczecin, Al. Powstańców Wielkopolskich 72, 70-111 Szczecin, Poland; 2 Pediatric, Adolescent Gynecology Clinic, Department of Gynecology, Endocrinology and Gynecological Oncology, Pomeranian Medical University in Szczecin, Unii Lubelskiej 1, 71-252 Szczecin, Poland; 3 Department of Perinatology and Gynecology, Poznan University of Medical Sciences, 60-535 Poznań, Poland; 4 Centers for Medical Genetics GENESIS, ul. Dąbrowskiego 77a, 60-529 Poznań, Poland; 5 II-nd Department of Ophthalmology, Pomeranian Medical University in Szczecin, Al. Powstańców Wielkopolskich 72, 70-111 Szczecin, Poland; 6 Department of Endocrinology, Metabolic and Internal Diseases, Pomeranian Medical University in Szczecin, Unii Lubelskiej 1, 71-252 Szczecin, Poland; 7 Department of Urology and Urologic Oncology, Pomeranian Medical University in Szczecin, Al. Powstańców Wielkopolskich 72, 70-111 Szczecin, Poland; 8 Clinic of Obstetrics and Gynecology, Institute of Clinical Medicine, Faculty of Medicine, Vilnius University, LT-08661 Vilnius, Lithuania

**Keywords:** T syndrome, hypogonadism, bone mineralization, muscle mass, muscle strength, hormone replacement therapy

## Abstract

Introduction: Patients with Turner syndrome (TS) often face skeletal and muscular challenges, including reduced bone mineral density (BMD) and muscle weakness. This comprehensive study sheds light on the complex interplay between muscle strength, BMD, and metabolic and endocrine parameters in TS and healthy subjects. Methods: A cross-sectional study involving 42 TS patients and 70 healthy women was conducted. All patients had their BMD determined in the L1–L4 lumbar spine section and in the whole skeleton as well as the parameters of body fat mass (BF), and visceral fat mass (VF) were also determined. The maximum gripping force was measured with a hydraulic manual dynamometer. In addition, a number of blood hormonal and metabolic parameters were determined. Results: In the TS group, hand grip strength correlated positively with triglyceride levels but not with BMD. Healthy individuals had a positive link between hand grip strength and BMD, while patients with TS did not show a significant association between the two. A trend suggested that longer recombinant human growth hormone (rhGH) therapy might improve BMD in the L1–L4 region. Multiple linear regression analysis revealed that muscle strength assessment may be a potential exponent of reduced BMD, and also used clinically in young adult women but not in individuals with TS. Conclusions: The relationship between BMD variables and hand grip might differ between the two groups, potentially indicating distinct musculoskeletal characteristics in TS patients. Longer rhGH therapy in TS patients may have a positive effect on BMD in the L1–L4 region. Understanding the intricate relationships between these factors is important for optimizing clinical management strategies and improving the quality of life for TS patients.

## 1. Introduction

Muscles and bones, traditionally seen as separate systems, are closely linked through mechanical signals and molecular communication [1]. Mechanical stress from muscle contractions during weight-bearing and resistance exercises triggers bone remodeling, while bones provide structural support for muscle attachment, enabling coordinated movements and skeletal integrity [1,2,3]. This interplay is a dynamic axis of physiological regulation driven by mechanical and hormonal factors.

Turner syndrome (TS), a genetic disorder characterized by partial or complete absence of one X chromosome in females, has significant implications for the musculoskeletal system. The genetic background of TS can vary and also determine the final phenotype of the patient. Roughly 40–50% of females exhibit monosomy X, 15–25% present 45,X/46,XX mosaicism, and ~3% show 45,X/46,XY karyotypes [4,5,6]. X chromosome structural anomalies occur in 20%, commonly featuring isochromosome Xq [4]. A minority of patients retain Y chromosome elements [5].

In Turner syndrome (TS), patients face skeletal and muscular challenges due to estrogen deficiency from ovarian insufficiency, leading to reduced bone density [7,8,9]. Estrogen replacement therapy (ERT) is common, but the influence of other endocrine factors like growth hormone (GH) and thyroid function on bone health requires further study [10,11,12]. TS individuals also contend with muscle issues, including weakness, limited functionality, and an increased risk of metabolic disorders [13,14]. Muscle weakness in TS results from hormonal imbalances, limited physical activity, and GH failure [15,16,17].

Patients with TS typically have GH insensitivity. Recombinant human GH (rhGH) treatment is recommended at higher doses than for simple GH deficiency [18]. rhGH positively influences bone mineralization by promoting bone growth and density [19]. It can also improve muscle strength by enhancing muscle development [20]. Individual responses to rhGH may vary, and treatment should be tailored and monitored by endocrinologist.

Individuals with TS may exhibit skeletal features such as short stature, cubitus valgus, a shield-shaped chest, scoliosis, pterygium colli, and short hand and foot bones. While these features are present, TS itself is not classified as a skeletal dysplasia [3,18].

Understanding the interplay among endocrine parameters, bone health, and muscle strength in TS is crucial for optimizing treatments and improving outcomes [21]. Identifying specific hormonal deficiencies affecting bone and muscle can guide personalized interventions, like hormonal treatments or tailored physical activity, to enhance the quality of life for TS patients [22]. Evaluating the effects of therapies like ERT and GH replacement on bone mineralization and muscle strength can inform clinical decisions on their effectiveness [18].

The main objective of this research endeavor is to conduct a comprehensive evaluation of the interaction between hormonal and metabolic parameters and their effects on handgrip strength in individuals with TS. Secondly, we sought to determine whether muscular grip strength has the same effect on BMD in both healthy patients and patients with TS. This could be helpful in planning personalized treatment strategies, such as hormonal interventions or choosing a specific type of physical activity, as well as to improve the quality of life in women with TS. In addition, we intend to investigate whether the duration of rhGH therapy affects handgrip strength, as well as BMD and BF in these individuals.

## 2. Materials and Methods

### 2.1. Subjects

This was a cross-sectional study conducted between March 2012 and October 2019 involving young patients with TS from a single university hospital referral center. The exclusion criteria were any chronic disease (except for autoimmune thyroiditis) or medication (except for GH and estrogen and levothyroxine) with known effects on muscle or bone. There were 42 patients with TS and 70 healthy women included in the study.

All patients with TS have stopped growing. Their karyotypes were either X monosomy (*n* = 21), the classical 45,X/46,XX mosaicism (*n* = 14), other double or triple cell line mosaics with at least two aberrant cell lines (*n* = 7). All girls with TS were treated with rhGH at an adjusted dose (1.2–2 mg/m^2^/day) according to the clinical response in the past. The median duration of the treatment with GH was 5.2 years (age range 0.8–14.5 years). Estrogen replacement was given to 35 patients to initiate puberty and to 3 patients for secondary ovarian failure, while 4 patients had signs of spontaneous pubertal development. Transdermal estrogen replacement began at a median age of 12.7 years (age range of 10.8–14.6 years) and lasted for an average of 2.5 years, with a starting dose of 17β-estradiol 0.00625 mg/day, gradually increasing to 0.05 mg/day. After a mean period of 2.5 years, the transdermal therapy was continued sequentially with 0.05 mg/day of estradiol for two weeks and 0.05 mg/day estradiol plus 0.17 mg/day noretysterone acetate for two weeks. Fifteen women in the study group and 4 in the control group were taking levothyroxine at a dose of 25 to 125 mcg for hypothyroidism. In all of them, thyroid stimulating hormone (TSH) and free thyrixin (FT4) were within the reference norm range.

The control group (CG) was constituted of 70 age-matched, healthy, premenopausal women who were recruited in the Outpatient Clinic of Endocrinology, Pomeranian Medical University in Szczecin, (Szczecin, Poland). All of the controls were Caucasian, had regular menstrual cycles, did not take any estrogen, progesterone or testosterone therapy, did not take any contraception, did not smoke or abuse alcohol and had not given birth in the past.

All patients underwent a standard history and physical examination; other data, such as the patient’s age, were collected from the patient’s registry data. Anthropometric measurements were taken (height was measured in a standing position, using a digital telescopic wall-mounted stadiometer and weight was determined to the nearest 0.1 kg using an electronic scale), on the basis of which the body mass index was calculated.

### 2.2. Biochemical Analyses

Fasting blood samples were coll1ected from all subjects included in the study during the follicular phase of the menstrual cycle (3rd, 4th, or 5th day of menstrual cycle). All tests were performed in the hospital’s prospective laboratory by standard assays (Luminex technology R&D System, Inc., Minneapolis, MN, USA; Beckman DXC 800, Danaher Corporation, Miami, FL, USA; Analox GM7 Analyzer, Analox Instruments, London, UK) at the study’s core laboratory of the Pomeranian Medical University in Szczecin.

#### 2.2.1. Assessment of Bone Mineralization

In all participants, BMD was determined in the L1–L4 section of the lumbar spine and in the whole skeleton using dual-energy X-ray absorptiometry (DXA- Dual-energy X-ray absorptiometry). The study was performed with a GE Lunar Prodigy Advance instrument (Madison, WI, USA) using enCORE software (version 8.8). Daily calibration using company-provided phantoms was performed. The results obtained were presented as absolute values (g/cm^2^) and in the form of an index Z-score comparing the result obtained in subject to a control group of the same age. A Z-score > (−1.0) was considered a normal value, according to current standards.

#### 2.2.2. Assessment of Body Composition

A body composition analysis of the study participants was performed using the DXA method. A GE Lunar Prodigy; Madison, WI, USA with CoreScan™ H8801CP version 14 automatic software and automatic whole body scanning method (Body Composition) using the original manufacturer’s software was used for the study. The parameters that were determined were fat mass (BF), and visceral fat mass (VF).

### 2.3. Muscle Function Assessment

The maximum gripping force was measured with SAEHAN 5030J1 hydraulic manual dynamometer using springs with a load of 40 kG. In the measurements carried out by this method, the value of isometric hand and forearm strength was obtained. The patients remained in a standing position during the study. The tested upper limb was bent at the elbow joint at 90 degrees with the arm remaining in contact with the torso. Each of the patients, after short explanation, was asked to squeeze the dynamometer bar three times with maximum power before a rest period of 30 s, both with their right and left hand. The average value of the three measurements was considered the proper measurement of muscle strength. The results were expressed in kilograms.

### 2.4. Statistical Analysis

Variables were presented as medians and standard deviations (SD). If assumptions for analysis of variance (ANOVA) were met, parameters were compared between the two groups (with or without Turner’s syndrome) using ANOVA, otherwise Kruskal–Wallis tests were used [11].

Pearson’s correlations (and therefore subsequently linear regressions) were only performed if three assumptions were met: no outliers (with Grubb’s test), no heteroscedasticity (Breusch–Pagan test), and bivariate normality (Shapiro–Francia test). Pearson correlation assumptions used a significance cut-off of *p =* 0.01. For linear regression four assumptions were tested: linearity (Rainbow test), normality of residuals (Shapiro–Wilk test) and homoscedasticity (Breusch–Pagan test) with a significance cut-off of *p* = 0.01 and multicolinearity with a variance inflation factor cut-off of 5. If all assumptions were met, then a first model was created with all variables and adjustors. A second model was created after step-wise removal of variables with *p*-values greater than 0.05.

All statistical analyses were performed using the R statistical platform (version 4.2.3, 2023; RRID:SCR_001905, https://cran.r-project.org, accessed on 4 September 2023).

## 3. Results

The individuals from both groups (TS and CG) were in comparable ages (*p* = 0.779) and the difference in their body weight was not statistically significant (*p* = 0.251) There was a significant difference in BMI and height between the groups (*p* < 0.01). The TS group tended to have higher values for BMI compared to the CG (Table 1).

Most of the clinical and laboratory characteristics (Table 2) show differences between the two groups. Notable differences include HDL, LDL, TG, FSH, testosterone, DHEA-S and cortisol, where *p*-values were less than 0.05, indicating significant differences between the TS group and the CG.

Table 3 mainly focuses on bone and muscle characteristics. The hand grip measurements (right, left, and mean) and various bone density measurements show significant differences between the two groups. Additionally, VF and some bone density parameters also differ significantly between patients with TS and healthy individuals.

The data in Table 4 reveals intriguing trends regarding the relationship between the duration of rhGH therapy in TS patients and their bone and muscle strength characteristics. While differences in hand grip strength were not statistically significant, it is important to note that the study included a relatively small number of participants. Nonetheless, a noteworthy trend suggests that longer therapy might lead to improved BMD in the L1–L4 region (*p*-value of 0.065).

The results of linear regression analysis from Table 5 illustrate hand grip mean versus hormonal and metabolic parameters, as well as bone characteristics in patients with TS and healthy individuals adjusted for height (Adj1), weight (Adj2), and age (Adj3) reveal varying associations between hand grip strength and health-related variables. In patients with TS, a significant positive association with higher HOMA IR values is observed, but not in the CG. In patients with TS, hand grip strength is significantly positively related to TG levels, indicating higher grip strength correlates with higher TG levels. This association is not observed in the CG. While no significant association exists between hand grip strength and visceral fat in patients with TS, a highly significant negative relationship is evident in the CG, signifying that higher grip strength is linked to lower visceral fat. In individuals with TS, there is no significant connection between hand grip strength and BMD-L1–L4. However, healthy patients show a significant positive relationship, indicating that individuals with higher grip strength tend to have greater BMD in the L1–L4 region. In patients with TS, hand grip strength does not significantly correlate with height. In the CG, a significant positive association is identified, suggesting that individuals with greater hand grip strength are taller.

**Figure 1:**
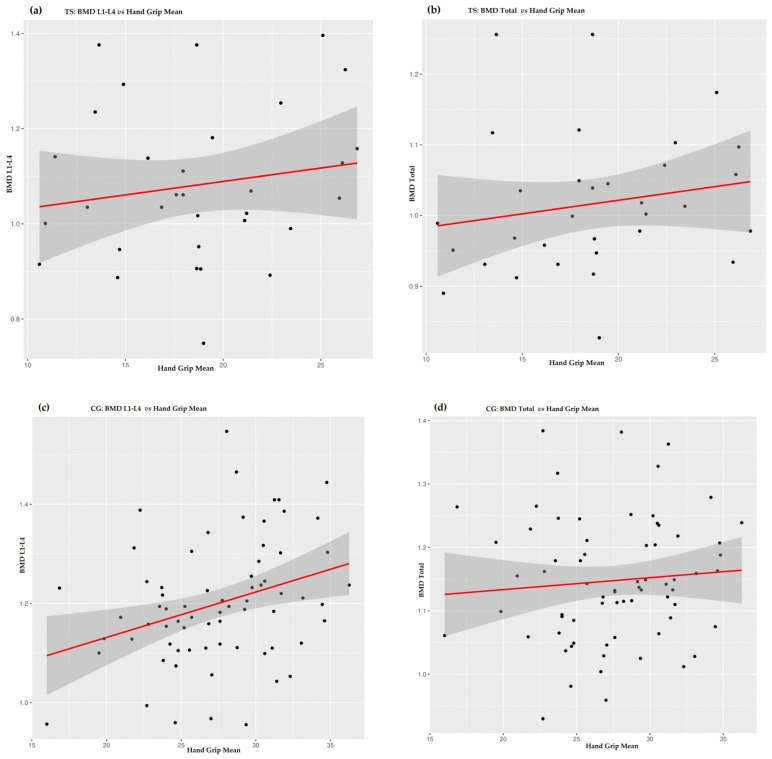
Linear regression (red lines; standard errors: dark grey) of bone mineral density parameters versus mean hand grip for patients with TS (**a**,**b**) and controls (**c**,**d**). (**a**) BMD L1–L4 in patients with TS; (**b**) BMD-total in patients with TS; (**c**) BMD L1–L4 in the control group (CG); (**d**) BMD-total in control group (CG).

There was no significant linear correlation between hand grip strength and BMD (both total BMD and BMD in the L1–L4 region) in individuals with TS, which differs from the relationship observed in the control group (Figure 1). This observation implies that the predictive ability of manual grip strength for BMD may be less robust in patients with TS than in healthy people, which may indicate hidden effects or compensatory mechanisms.

## 4. Discussion

Our findings reaffirm the well-established observation of diminished BMD and heightened osteoporosis risk among individuals with TS. These effects are primarily attributed to estrogen deficiency resulting from ovarian insufficiency [23,24,25]. Although serum estradiol levels in patients in the two groups did not differ significantly, it should be noted that measurements were taken in the early follicular phase. In healthy women with regular ovulatory cycles, estradiol levels are lowest in the early phase of the menstrual cycle. In patients with TS, serum estradiol concentrations mainly reflect drug absorption and are expected to remain relatively constant during follow-up. Estrogens play a crucial role in bone health by enhancing the activity of osteoblasts, stimulating the synthesis of various bone-related proteins, and inhibiting bone resorption by osteoclasts [26]. Similarly, androgens are also important for bone health, as they stimulate the proliferation and maturation of osteoblasts and influence the synthesis of key bone-related proteins [27]. Gravholt’s study in 1999 found reduced concentrations of androstenedione, free testosterone, sex hormone-binding globulin (SHBG), and dihydrotestosterone (DHT) by 25% to 40% in patients with TS [28]. Our study also showed lower testosterone concentrations compared to the control group.

In addition to bone health, we observed significantly weaker hand grip strength in females with TS compared to controls, which is consistent with the results of several previous studies [29,30]. This reduced muscle strength is a consistent feature of TS and is often attributed to hormonal imbalance and GH insufficiency/insensitivity, which can lead to reduced muscle mass and impaired neuromuscular function [21,22]. As well as the insufficiency of GH can hinder the development of muscle, making it more challenging for individuals to build and maintain muscle throughout their lives [21]. In our study, we found no correlation between final height and gripping strength in patients with TS, in contrast to a control group where hand grip strength was significantly greater in taller individuals. Given the clinical implications of reduced muscle strength, interventions targeting muscle function in patients with TS, such as resistance training, should be promoted [29,30].

During childhood and adolescence, the skeletal structure undergoes constant changes to meet increasing mechanical demands [31,32]. When considering the causes of reduced BMD in people with TS, it is important to distinguish between the direct effect of the absence of the X chromosome on bone metabolism and the different mechanical stimulation of bone by the muscular system [33]. Muscle contractions impose much greater mechanical loads on bones than gravitational forces alone. Reduced muscle mass, muscle weakness, and inadequate muscle tone can significantly affect the physical well-being of people with TS [9], while reduced bone mass can result from both reduced mechanical demands and inadequate adaptation to these demands [34]. In our study, we found no significant linear correlation between hand grip strength and BMD indices in women with TS, in contrast to a significant positive linear relationship in healthy subjects. While some studies have highlighted the role of rhGH therapy in improving muscle strength [35,36], others have emphasized the potential benefits of physical therapy and exercise [30,37,38]. Comparison of our results with previous studies underscores the need for a personalized approach to address muscle strength deficits in patients with TS.

Analyzing the results of our study, we observed a negative effect of visceral adipose tissue on hand grip strength in the control group, which could not be proven in subjects with TS. Excess body fat, especially visceral fat, is associated with insulin resistance and systemic inflammation, which can impair muscle function [39,40]. In the context of TS, understanding the role of visceral adipose tissue in muscle weakness is crucial, as it can provide insight into strategies to improve muscle strength through weight control and dietary interventions [41].

Patients with ovarian dysgenesis due to TS have an increased risk of metabolic disorders, including insulin resistance and diabetes [42,43]. Elevated cholesterol levels in childhood promote the onset of atherosclerosis in adulthood [44]. Metabolic problems in patients with TS, such as insulin resistance, may contribute to increased mortality [45]. Some evidence suggests that metabolic disorders are associated with monosomy of the X chromosome [46]. The development of insulin resistance is influenced by body weight, body composition and karyotype [47,48]. It has been shown that normal-weight women with TS have a lower metabolic risk, potentially related to Fibroblast Growth Factor 21 (FGF 21) metabolism [42]. FGF21 significantly influences metabolism by enhancing insulin sensitivity, regulating glucose uptake, promoting lipid metabolism, increasing energy expenditure, and inducing the browning of white adipose tissue. In our study, we found similar glucose and insulin levels compared to control subjects, but triglyceride levels were higher in patients with TS. rhGH therapy may affect body composition and the development of insulin resistance [49], while the effect of estrogen on glucose metabolism is multifaceted, with conflicting results [50]. A recent meta-analysis in estrogen-deficient menopausal women showed a beneficial effect of low-dose estrogen replacement therapy on lipid profiles [51].

Our results also indicate a positive trend between the duration of rhGH therapy and BMD in the L1–L4 region. Although the *p*-value does not reach statistical significance, the results suggest a potential effect of longer rhGH therapy on improving BMD in this region. Our results are in line with the work of Aycan et al. who found that short-term GH therapy in TS patients has no significant effect on bone density measured mainly at the beading sites [52]. Sass et al. reported that BMD increased during treatment with rhGH at a dose of 2 mg/m^2^/day combined with low doses of estrogen [53]. However, these findings underscore the need for further investigation in larger cohorts to better understand the impact of rhGH therapy on musculoskeletal health in patients with TS.

Although our results on hand grip strength in relation to the duration of rhGH therapy were not statistically significant, it should be noted that the study was conducted on a relatively small number of subjects. This underscores the need for further exploration of the cause of persistent muscle weakness in TS, which may be attributed to hormonal imbalance and GH deficiency [7,54].

This study exhibits several strengths, including a detailed methodology covering diverse assessments, the inclusion of an age-matched control group, and comprehensive data collection on clinical, laboratory, and musculoskeletal parameters. The rigorous statistical analysis enhances the study’s credibility. Moreover, the study’s clinical relevance lies in its exploration of the impact of hormone therapy on BMD in individuals with TS.

However, certain limitations need consideration. The retrospective design introduces potential bias, and the small sample size limits the generalizability of findings. The focus on grip strength leaves other essential factors relatively unexplored. Challenges in data analysis, such as missing data and potential confounding factors, may impact the accuracy of results. The single-center nature of the study might limit population diversity, and the limited exploration of exercise impact on muscle strength poses a gap in understanding.

## 5. Conclusions

This study underscores the intricate interplay between muscle strength and hormonal/metabolic parameters in TS. VF emerges as a negative influence on hand grip strength, emphasizing the importance of weight management. The lack of correlation between height and muscle strength highlights the need for personalized interventions. While prolonged rhGH therapy did not significantly impact grip strength, a potential positive effect on BMD in the L1–L4 region was suggested. Recognizing these complexities is essential for tailoring effective strategies to address the unique challenges faced by individuals with TS.

## Figures and Tables

**Table 1:** Demographic and anthropometric characteristics in patients with TS (TS) and control group (CG).

Characteristic	TS; SD; *n* = 42	CG; SD; *n* = 70	*p*-value
age [years]	23.7; 2.01	23.1; 1.57	0.779
BMI ^1^ [kg/cm^2^]	26.9; 3.67	22.4; 4.40	<0.01
height [cm]	159; 4.71	168; 5.60	<0.01
weight [kg]	61.9; 7.53	63.9; 12.3	0.251

^1^ BMI—body mass index.

**Table 2:** Clinical and laboratory characteristics of patients from the TS (TS) group and the control group (CG).

Characteristic	TS; SD (*n*)	CG; SD (*n*)	*p*-value
Glucose [mg/dL]	87.7; 8.74 (42)	89.6; 6.75 (70)	0.297
Insulin [mU/mL]	12.4; 5.02 (42)	10.9; 6.12 (70)	0.102
HOMA-IR	2.68; 1.15 (42)	2.45; 1.52 (70)	0.218
25-hydroxyvitamin D	29.4; 5.43 (40)	27.7; 6.5 (70)	0.310
Total cholesterol [mg/dL]	170; 52.6 (40)	181; 27 (70)	0.261
HDL [mg/dL]	53.6; 13.5 (40)	71; 18.3 (70)	<0.01
LDL [mg/dL]	132; 34.5 (40)	104; 22.9 (70)	<0.01
TG [mg/dL]	118; 52.3 (40)	83; 32.1 (70)	<0.01
Ca total [mmol/L]	2.41; 0.159 (41)	2.42; 0.077 (64)	0.067
Ca ion [mmol/L]	1.26; 0.027 (32)	1.27; 0.0393 (63)	0.348
TSH [μIU/mL]	2.95; 1.51 (38)	2.17; 1.05 (70)	0.065
FT4 [pg/mL]	1.3; 0.191 (38)	1.23; 0.16 (66)	0.119
FSH [mIU/mL]	42.5; 28.1 (39)	6.06; 1.88 (70)	<0.01
Estradiol [pg/mL]	45.1; 29.6 (39)	38.7; 25.6 (70)	0.863
Prolactin [ng/mL]	21.1; 12.2 (38)	18.7; 10.8 (70)	0.337
Testosterone [ng/mL]	0.267; 0.224 (38)	0.361; 0.159 (70)	0.032
DHEA-S [μg/mL]	157; 77.3 (42)	258; 120 (66)	<0.01
Cortisol total [μg/mL]	13.2; 5.27 (39)	18.8; 7.68 (63)	<0.01
ACTH [pmol/L]	14.9; 7.11 (35)	16.9; 6.1 (60)	0.064

Abbreviations: HOMA-IR- homeostatic model assessment for insulin resistance; LDL—low-density lipoprotein; HDL—high-density lipoprotein; TG—trigliceride; Ca—calcium; TSH—thyroid-stimulating hormone; FT4—free thyrixin; FSH—follicle-stimulating hormone; DHEA-S—dehydroepiandrosterone sulfate; ACTH—Adrenocorticotropic hormone.

**Table 3:** Bone and muscle strength characteristics in patients with TS and the control group.

Characteristic	TS; SD; *n* = 42	CG; SD; *n* = 70	*p*-value
Hand grip—left [kg]	19.1; 4.68	28.3; 4.67	<0.01
Hand grip—right [kg]	18.3; 4.93	26.7; 4.85	<0.01
Hand grip mean [kg]	18.7; 4.65	27.5; 4.40	<0.01
BF [kg]	22.4; 10.60	20.7; 9.55	0.619
VF [kg]	0.61; 0.46	0.39; 0.21	<0.01
BMD Total [g/cm^2^]	1.02; 0.11	1.15; 0.10	<0.01
BMD Total (Z-Score)	−0.2; 1.2	0.2; 0.9	<0.01
BMD L1–L4 [g/cm^2^]	1.08; 0.16	1.2; 0.13	<0.01
BMD L1–L4 (Z-Score)	−0.4; 1.1	0.1; 0.9	<0.01

Abbreviations: BF—body fat; VF—visceral fat, BMD—bone mineral density.

**Table 4:** Bone and muscle strength characteristics in patients with TS depending on the time of rhGH therapy in the past.

Characteristic	TS rhGH < 2 years; SD; *n* = 12	TS rhGH ≥ 2 years; SD; *n* = 30	*p*-value
Hand grip—left [kg]	18.2; 4.28	20.1; 4.17	0.084
Hand grip—right [kg]	18.0; 4.02	19.6; 4.92	0.134
Hand grip mean [kg]	18.4; 4.15	19.5; 4.41	0.103
BF [kg]	22.6; 10.68	22.2; 9.13	0.619
VF [kg]	0.63; 0.49	0.60; 0.41	0.095
BMD Total [g/cm^2^]	1.01; 0.098	1.04; 0.0982	0.621
BMD Total (Z-Score)	−0.2; 1.2	−0.2; 0.9	0.892
BMD L1–L4 [g/cm^2^]	1.07; 0.12	1.1; 0.13	0.172
BMD L1–L4 (Z-Score)	−0.4; 1.1	−0.2; 0.9	0.065

Abbreviations: BF—body fat; VF—visceral fat, BMD—bone mineral density.

**Table 5:** Linear regression results for hand grip mean versus hormonal and metabolic parameters as well as bone characteristics in patients with TS (TS) and healthy individuals (CG) (adjusted for height-Adj1, weight-Adj2 and age-Adj3).

Variable 1 = Hand grip mean							
Variable 2	Group	Variable 2 *p*-value	Linear Model Estimate	Estimate Standard Error	R-Squared	Beta Coefficient	Beta Standard Error
HOMA-IR	TS	0.0388 * ^abc^	1.60	0.738	0.209	n.d.	n.d.
	CG	0.152 ^abc^	−0.534	−0.534	0.0573	n.d.	n.d.
TG [mg/dL]	TS	0.0164 * ^n^	0.0375	0.0167	0.0755	0.434	0.170
	CG	0.272 ^abc^	−0.0188	0.0170	0.0678	n.d.	n.d.
VF [kg]	TS	0.216 ^abc^	0.00237	0.00187	0.123	n.d.	n.d.
	CG	0.000139 *** ^b^	−0.00832	0.00242	0.196	−0.747	0.185
BMD L1–L4 [g/cm^2^]	TS	0.497 ^abc^	3.82	5.54	0.0866	n.d.	n.d.
	CG	0.00622 **	12.0	4.14	0.163	0.324	0.115
Height	TS	0.269 ^bc^	−0.204	0.181	−0.0291	n.d.	n.d.
	CG	0.0476 * ^n^	0.180	0.0893	0.0432	0.239	0.119

Adjustments: a = heigth, b = weight, c = age; n = no adjustments; n.d. = not determined; Significance levels: *** *p* < 0.001, ** *p* < 0.01, * *p* < 0.05; all data given to 3 significant figures. Abbreviations: HOMA-IR—homeostatic model assessment for insulin resistance; TG—triglyceride, VF—visceral fat, BMD—bone mineral density.

## Data Availability

Data are available on special request after contacting author (M.K.).
